# The effect of crRNA–target mismatches on cOA-mediated interference by a type III-A CRISPR-Cas system

**DOI:** 10.1080/15476286.2022.2150812

**Published:** 2022-11-24

**Authors:** Mohamed Nasef, Sarah A. Khweis, Jack A. Dunkle

**Affiliations:** Department of Chemistry and Biochemistry, University of Alabama, Tuscaloosa, AL, USA

**Keywords:** CRISPR-Cas, crRNA, Cas10-Csm, cyclic oligoadenylates, bacterial immunity

## Abstract

CRISPR systems elicit interference when a foreign nucleic acid is detected by its ability to base-pair to crRNA. Understanding what degree of complementarity between a foreign nucleic acid and crRNA is required for interference is a central question in the study of CRISPR systems. A clear description of which target–crRNA mismatches abrogate interference in type III, Cas10-containing, CRISPR systems has proved elusive due to the complexity of the system which utilizes three distinct interference activities. We characterized the effect of target–crRNA mismatches on in vitro cyclic oligoadenylate (cOA) synthesis and in vivo in an interference assay that depends on cOA synthesis. We found that sequence context affected whether a mismatched target was recognized by crRNA both in vitro and in vivo. We also investigated how the position of a mismatch within the target–crRNA duplex affected recognition by crRNA. Our data provide support for the hypothesis that a Cas10-activating region exists in the crRNA–target duplex, that the Cas10-proximal region of the duplex is the most critical in regulating cOA synthesis. Understanding the rules governing target recognition by type III CRISPR systems is critical: as one of the most prevalent CRISPR systems in nature, it plays an important role in the survival of many genera of bacteria. Recently, type III systems were re-purposed as a sensitive and accurate molecular diagnostic tool. Understanding the rules of target recognition in this system will be critical as it is engineered for biotechnology purposes.

## Introduction

CRISPR-Cas systems provide prokaryotes an adaptive immune defence against bacteriophage and mobile genetic elements by utilizing a crRNA to detect foreign genes and Cas proteins to elicit an interference response against them[1], [2]. CRISPR-Cas systems can be organized into two classes and six types. In Class 1 systems, a crRNA in the presence of a multi-protein complex is used for interference, while in Class 2 systems, a single polypeptide bound to a crRNA carries out interference [3]. The subdivision of CRISPR systems into six types is based upon the presence of a signature Cas effector in each type, a protein which is structurally and evolutionarily unique to the type. The opportunistic pathogen, *Staphylococcus epidermidis* strain RP62A, possesses a CRISPR system categorized as type III-A due to the presence of the Cas10 protein, the signature of type III systems and the Csm2 protein which groups the system with III-A members. Type III systems are notable for their elaborate and multi-faceted interference activities.

**Figure 1.**
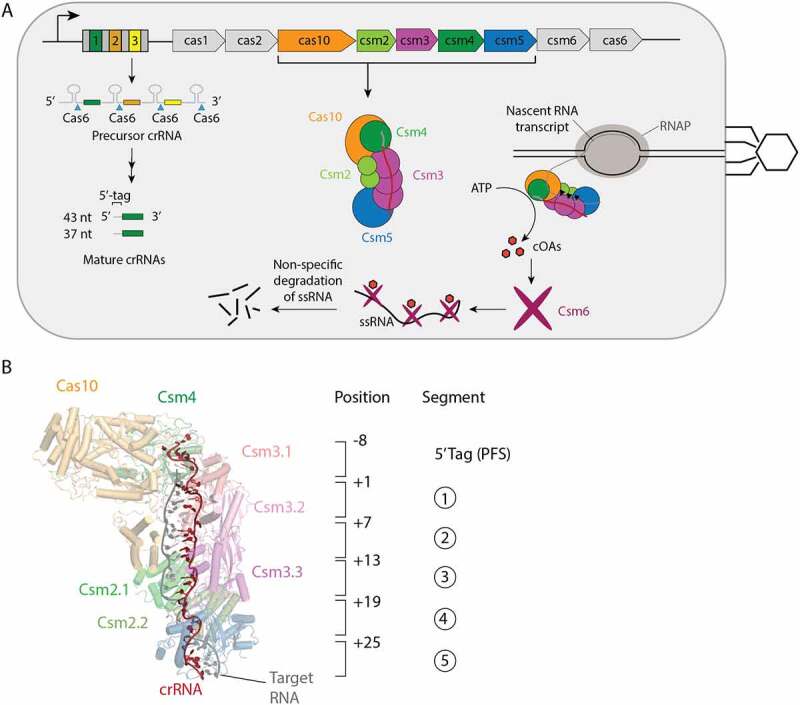
Interference mediated by the *S. epidermidis* Cas10-Csm complex. (A) The type III-A CRISPR-Cas locus in *S. epidermidis* contains a repeat-spacer region with three spacers (coloured) and four repeats (grey) and nine CRISPR-associated genes. The repeat-spacer is transcribed and processed during crRNA biogenesis into mature crRNA of primarily 37 and 43 nucleotides. The crRNA combine with five Cas proteins to form the Cas10-Csm effector complex which carries out interference against foreign genetic elements. An active site in the Csm3 protein cuts bound target RNA (black triangles) and Csm6, once stimulated by cyclic oligoadenylates (cOAs) indiscriminately cuts RNA inducing cell dormancy. Cas10 also possesses a ssDNA cutting activity that is not pictured. (B) The 8 nucleotides on the 5’ end of crRNA are referred to as the 5’ tag or the proto-spacer flanking sequence (PFS) and are not involved in base-pairing to cognate targets. The first nucleotide following the 5’ tag can engage in base-pairing to a cognate target and is numbered +1. Each six nucleotides of crRNA behaves as a structural unit with five nucleotides base-pairing and the sixth unpaired. This pattern repeats throughout the crRNA–target duplex and each six-nucleotide segment can be assigned a number 1–5.

The *S. epidermidis* CRISPR locus encodes a multi-protein Cas effector complex made up of the proteins Cas10, Csm2, Csm3, Csm4 and Csm5 bound to a crRNA originating from one of the three spacer genes of the locus (Fig. 1A) [4]. The Cas10-Csm complex surveils the cell for foreign RNA able to base-pair to its crRNA and upon its detection activates three interference activities: a single-stranded DNA cleavage activity in the HD domain of Cas10, a cyclic oligoadenylate synthesis (cOA) activity originating from the Palm2 domain of Cas10 and cleavage of the bound foreign RNA via an active site in the Csm3 protein [2]. The cOAs, 3-mer to 6-mer oligomers of adenosine monophosphate whose tail and head are bonded to form a cyclic structure, act as second messenger molecules binding to the Csm6 RNase and activating it to indiscriminately cleave cellular RNA driving the cell into dormancy [5–8]. The relative importance of the three interference activities in Cas10 systems has been debated and may be context dependent [9–13].

When a cognate target base-pairs to crRNA, by convention the base pair involving the first crRNA nucleotide after the 5’ tag region, is denoted as the +1 pair with subsequent pairs counting up from this position towards the 3’ end of crRNA (Fig. 1B). Structurally, each six nucleotides of the crRNA–target duplex form a discrete unit: five nts engage in base-pairs with A-form geometry and the sixth nt is flipped out of the stack and unpaired. The pattern of five-paired nts and an unpaired sixth nt continues throughout the crRNA–target duplex and therefore each six nt unit can be termed a segment.

Since the crucial first step in interference in all CRISPR systems is the recognition of a foreign gene by its base-pairing with cRNA, understanding the extent of base-pairing necessary for interference is a central question for all systems. For Cas9 and Cas12 systems, which are used extensively in genome editing, the question of target specificity takes on great significance because off-target gene cuts are deleterious to the precision of genome editing and therefore have been investigated extensively [14–18]. The effect of mismatches on interference by type III systems has been tested in microbiological assays that capture the effect in the complex context of an attack on the cell [19–21]. These assays, however, do not indicate to what degree each of the distinct interference activities is affected by mismatches since their individual contributions to interference are difficult to disentangle. Using in vitro biochemistry methods, the effect of mismatches on the DNA cleavage activity of Cas10 and the Csm3/Cmr4-mediated target RNA cutting activity has each been measured [22–25]. However, previous studies on the effect of mismatches on cOA synthesis were limited in scope not thoroughly addressing the effect of mismatch position within the crRNA–target duplex and the potential effect of sequence context [7,25–27]. To address these shortcomings, we investigated the effect of single mismatches across thirty nucleotides of the crRNA–target duplex in two sequence contexts. In vitro assays provided evidence for a Cas10-activating region, a location in the duplex with heightened sensitivity to mismatches; however, these mismatches minimally affect the affinity of target binding to cRNA [25]. We performed a cell-based transformation efficiency assay that depends on cOA synthesis. This assay allowed us to compare the effects of mismatches on cOA synthesis in vitro with their effect on cOA-driven interference in cells. The cell-based assay did not display the strong positional effects seen in vitro but confirmed the finding that sequence context is an important variable affecting how Cas10-Csm responds to mismatches.

## Results

**Figure 2.**
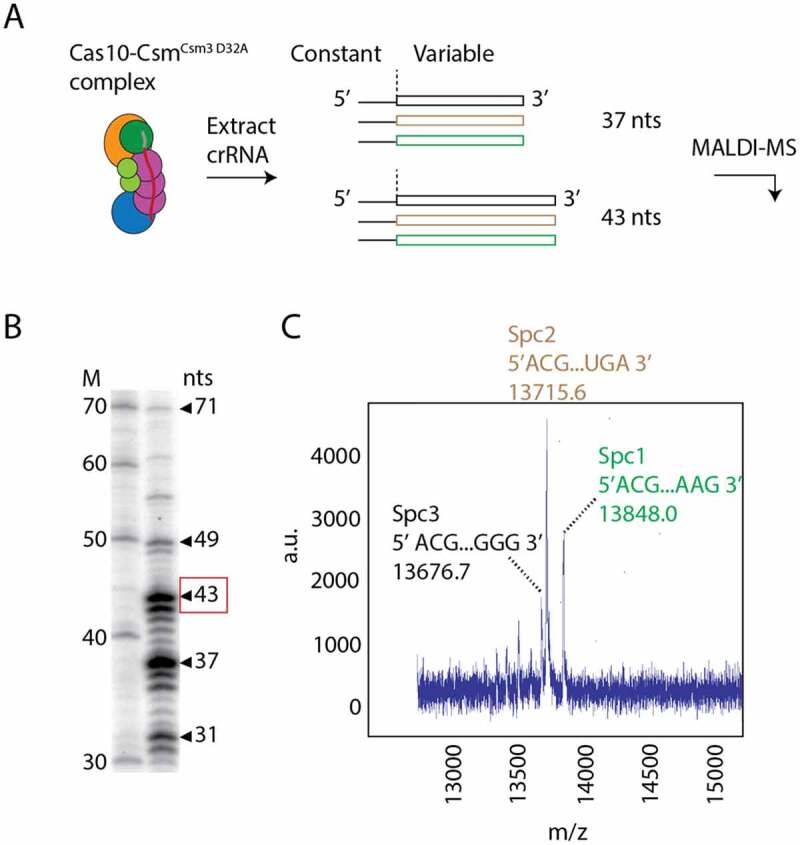
Identification of crRNAs bound to Cas10-Csm by mass spectrometry. (A) Cas10-Csm^Csm3 D32A^, a variant of the complex that is without Csm3 catalysed target RNase activity, co-purifies from *S. epidermidis* cells bound to crRNAs derived from three spacers with lengths of predominantly 37 and 43 nucleotides. The 5’ tag region of crRNA has a constant sequence derived from the repeat regions that flank spacers in the genome. Spacer regions in the CRISPR-Cas loci encode for unique crRNAs of variable sequence. (B) ^32^P-labelled, extracted crRNAs were visualized on a urea-PAGE gel revealing a distribution of intermediate and mature species with the 37 and 43 nts being most prominent. The 43-mer crRNA band, for which mass spectrometry data is shown, is highlighted with a red box. (C) MALDI mass spectrometry performed on the mixture of crRNAs identifies distinct peaks associated with the unique sequence of each RNA.

Previously, we described a method for purification of the Cas10-Csm effector complex from *S. epidermidis* cells that is active in cOA synthesis [7]. The method utilizes the *pcrispr* plasmid wherein the *S. epidermidis* CRISPR-Cas10 locus has been relocated from genomic DNA and *csm2* has been fused to a hexahistidine tag. We used immobilized metal affinity chromatography (IMAC) followed by ultracentrifugation to separate, from other cellular components, the ribonucleoprotein complex which is expected to contain a mixture of the three crRNA species. To confirm that purified Cas10-Csm complexes contain the expected crRNAs, we extracted cRNAs from the Cas10-Csm complex with phenol–chlorofom and analysed them by urea-PAGE and matrix-assisted laser desorption ionization mass spectrometry (MALDI-MS) (Fig. 2). Fig. 2B shows a distribution of crRNA lengths consistent with previous analysis of *S. epidermidis* Cas10-Csm with 37 nts and 43 nts as the most abundant lengths. MALDI-MS of the extracted cRNAs allows precise measurement of molecular mass and the unique nucleotide sequences of each crRNA should produce a unique *m*/*z* signature (Fig. 2C). For example, in the family of peaks corresponding to the 43-nt crRNAs we observed three peaks by MALDI-MS each with an *m*/*z* value within 0.3 to 2.5 *m*/*z* units of the value predicted by the gene sequence of each spacer (Table S1). These data confirmed that the Cas10-Csm we isolated possessed crRNAs targeting the nickase *(Nes)* mRNA (targeted by *spc1*) and the *Cn20* mRNA (targeted by *spc2*) which we intended to use as model targets for interference.

### Mismatches proximal to Cas10 can reduce cOA synthesis

**Figure 3.**
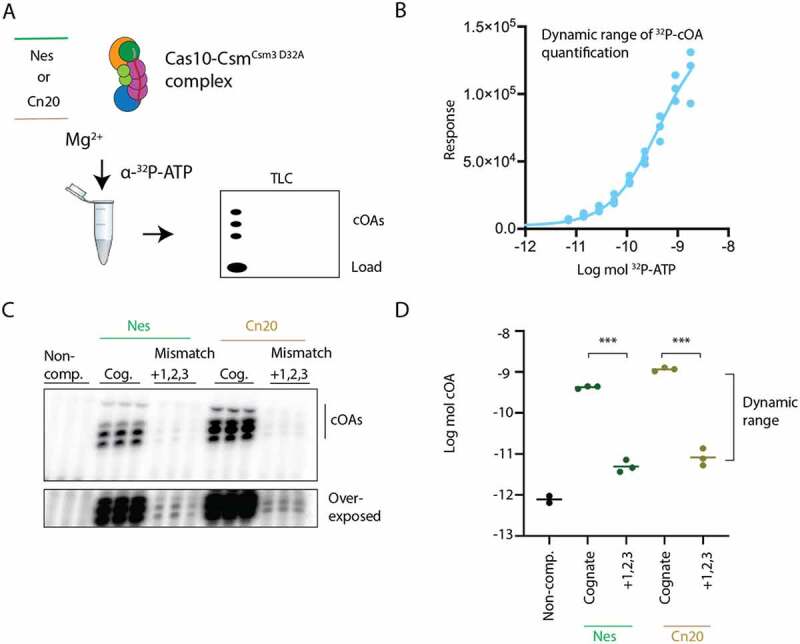
A triple mismatch in segment 1 of the crRNA–target duplex inhibits cOA synthesis. (A) cOA synthesis was assayed by incubating either cognate target–RNAs or RNAs containing mismatches at positions +1, +2 and +3 with Cas10-Csm complex and α-^32^P-ATP. Radiolabeled cOA products were separated on a TLC plate and visualized by phosphorimaging. (B) A serial dilution of known amounts of ^32^P-ATP was applied to a TLC plate and quantitated to establish the dynamic range of the assay. (C) An image of cOA synthesis reactions, performed in triplicate, in the presence of cognate (Cog.) target–RNA or triple mismatch target–RNA. Data are shown for target RNAs complementary to the spacer-1 crRNA (*Nes*) and the spacer-2 crRNA (*Cn20*). The lower image shown is the same TLC plate overexposed. (D) Reactions shown in (C) were quantified using ImageJ and interpolated using the curve in (B) to moles of product generated. ***P <0.001.

Next, we sought to develop a quantitative assay for cOA synthesis by *S. epidermidis* Cas10-Csm that could conveniently analyse the results of dozens of reactions in a few hours. To this end, we adapted a published protocol for separation of cOA products from the α-[28]P-ATP reactant by thin layer chromatography (Fig. 3A) [28]. To gauge the dynamic range of the assay, we performed titrations of α-^32^PATP in triplicate, spotted the solutions on the TLC plates and integrated the phosphorimaging results (Fig. 3B). Interpolation of the dose–response curve demonstrated that the technique had a dynamic range of approximately two orders of magnitude. We had previously shown that when Cas10-Csm was bound to a *Nes* target with mismatches in segment 1, which was recently termed the Cas10-activating region, dramatically lower amounts of cOA were produced [7,25]. Therefore, we reasoned that three consecutive mismatches in segment 1 would inhibit in vitro cOA synthesis by Cas10-Csm (Fig. 3C). The Csm3 protein of Cas10-Csm contains a divalent metal dependent active site that cleaves bound target RNA thereby inhibiting cOA synthesis [7]. Since this activity would likely be affected by the mismatches and make it difficult to resolve the effect of mismatches solely on activating Cas10 for cOA synthesis, we performed our assays with complex in which the Csm3 protein possesses the site-directed mutant D32A (Cas10-Csm^Csm3 D32A^). We assayed the effect of a triple mismatch in segment 1 in two sequence contexts, feeding Cas10-Csm a synthetic RNA mimicking the *Nes* transcript with three mismatches or a synthetic RNA mimicking the *Cn20* transcript with three mismatches. In both cases, only, an extremely small amount of cOAs were produced an amount at the very low end of the dynamic range of our assay. We conclude that a triple mismatch in segment 1 has at least a two orders of magnitude effect on cOA synthesis in vitro.

**Figure 4.**
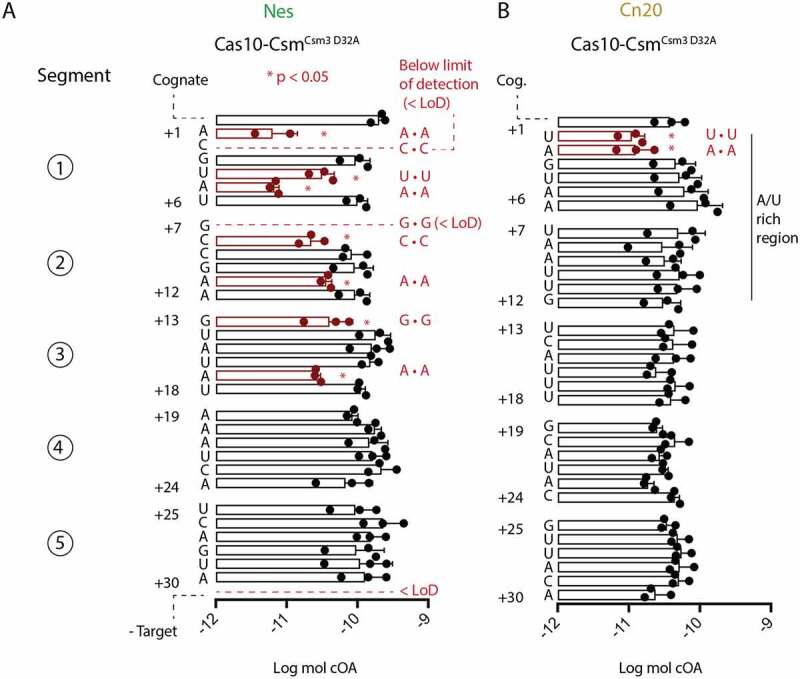
Single mismatches in the crRNA–target duplex inhibit cOA synthesis in a position- dependent and sequence-dependent manner. (A) Triplicate cOA synthesis reactions were performed with *Nes* target RNAs containing a single mismatch at position +1 to position +30 in the presence of α-^32^P-ATP and the cOA products were resolved by TLC and quantitated by phosphorimaging. Targets which produce cOA levels significantly different than cognate target RNA are shown in red. Mismatches at +2 or +7 or reactions conducted without any target were below the level of detection (<LoD). Mismatches significantly different than cognate have the identity of the mismatch given (for example A • A indicates two adenosines mismatched with each other). (B) Triplicate cOA synthesis reactions with *Cn20* target RNAs containing single mismatches from position +1 to position +30. As above, targets which produce cOA levels significantly different than cognate target RNA are shown in red. *P < 0.05.

We next sought to characterize across the length of the crRNA–target duplex how more subtle, single mismatches may affect cOA synthesis. A library of RNAs mimicking the *Nes* and *Cn20* transcripts was produced by in vitro transcription from synthetic DNA duplexes coding for each transcript (Fig. S3). We systematically introduced a single mismatch into each position from +1 to +30 of both the *Nes* and *Cn20* transcripts by changing each transcript nucleotide to its Watson–Crick base-pair partner and fed these targets to Cas10-Csm^Csm3 D32A^ to initiate cOA synthesis (Fig. 4). The products of three technical replicates were separated by TLC and quantitated (Figs. S4, S5). For the *Nes* transcript, we identified nine positions for which there was a statistically significant reduction in cOA synthesis compared to a cognate target (Fig. 4A). These positions, all occurring in segments 1–3, are biased towards the 5’ end of the crRNA–target duplex, in and near the region termed the Cas10-activating region [25]. Two *Nes* mismatches produced cOA levels equivalent to our no-target negative control, amounts below our level of detection, the +2 and +7 mismatched positions (Fig. 4A). We characterized the effect of single mismatches across the length of the *Cn20* transcript and observed a different pattern. In the *Cn20* sequence context, two mismatches at positions +1 and +2 had a statistically significant reduction in cOA levels, but the amount of the reduction was about fivefold, less than the dramatic decreases seen in some *Nes* mismatches (see *Nes* positions +1, +2, +5 and +7) (Fig. 4A, 4B). However, in keeping with the pattern observed for *Nes* mismatches, the only *Cn20* mismatches which reduced cOA synthesis were in the Cas10-activating region. The segmented 6 nt structure of the crRNA–target duplex described previously dictates that each sixth nt is not base-paired. Therefore, introduction of a mismatch into position +6, +12, +18 or +24 is not expected to have an effect on cOA synthesis and indeed no statistically significant differences between these mismatched positions were observed in either the *Nes* or *Cn20* context, a powerful internal control in our experiment. An additional control experiment was performed: for mismatch positions in the Cas10-activating region with large effects on cOA synthesis, we repeated the cOA synthesis experiment with synthetic target RNAs to ensure that it was not heterogeneity in the IVT RNAs producing the synthesis defect (Fig. S6). Using the TLC-based assay, we observe varying absolute amounts of cOA produced from experiment to experiment and between *Nes* targets versus *Cn20* targets. This can be observed in the log moles cOA in Figs. 3, 4 and S6. The cause of this difference is unclear but appears to be a systematic difference and therefore does not affect our main goal to identify large differences in the effect of mismatches on cOA synthesis within a particular target RNA. Our studies affirm that the Cas10-activating region of the crRNA–target duplex plays a special role in stimulating cOA synthesis but demonstrate that sequence context also matters: the effect of a single mismatch on cOA synthesis is determined by position within the duplex and some contribution from local sequence context. We speculate that local GC content is the relevant parameter.

### The binding affinity of mismatched targets is minimally affected

**Figure 5.**
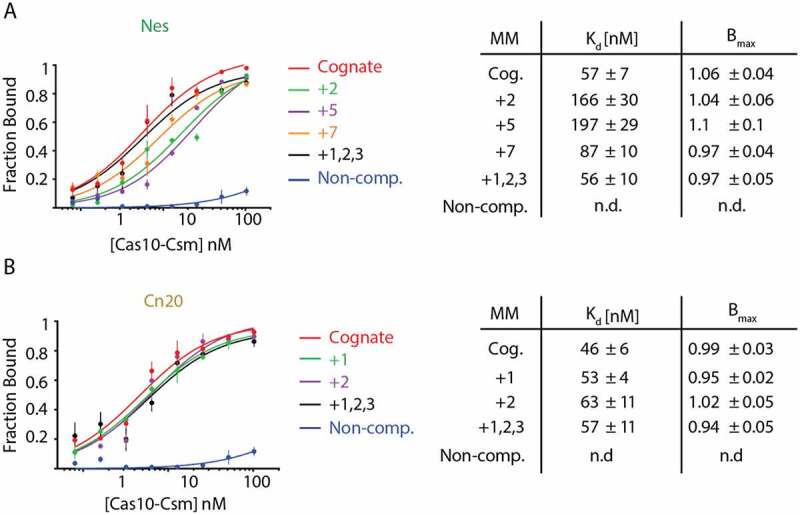
Single and triple mismatch target–RNAs show only modest changes in binding affinity towards Cas10-Csm^Csm3 D32A^ compared to cognate targets. (A) ^32^P-labelled *Nes* targets were incubated with increasing amounts of Cas10-Csm and bound target was isolated with a nitrocellulose and Hybond double membrane assay. Fraction bound was found by quantitating the amount of ^32^P-target on the nitrocellulose membrane (Cas10-Csm bound) versus the total amount of target (nitrocellulose plus Hybond) by phosphorimaging. (B) An affinity binding assay was also performed with ^32^P-labelled *Cn20* targets.

Two potential mechanisms could explain the decrease in cOA synthesis caused by mismatches at some positions within the crRNA–target duplex: either the mismatch fails to induce a conformational change in Cas10-Csm necessary for cOA synthesis or the mismatch causes a dramatic decrease in binding affinity of the target for Cas10-Csm. To test the latter mechanism, we performed an affinity binding assay comparing the mismatches to cognate target RNA (Fig. 5). We used synthetic RNAs mimicking mismatched transcripts of interest, those with the largest cOA synthesis defects. Some mismatches in the *Nes* transcript displayed a modest increase in the K_d_ for binding to Cas10-Csm; however, the approximately threefold differences observed for +2 and +5 mismatched transcripts would have a modest effect on the amount of target bound in the cOA synthesis assay since the target was present at 400 nM, enough to still drive binding of most transcript to the effector complex. The *Nes* +7 and +1,2,3 (triple) mismatched transcripts did not show a meaningful change in K_d_ and neither did any of the *Cn20* mismatched transcripts. This was expected since when Cas10-Csm is bound to a 37 nt cRNA, five segments of crRNA–target can bind, and in this context, a loss of one to three base-pairs is unlikely to dramatically alter the K_d_ of target for the crRNA bound Cas10-Csm. Since binding affinity is not dramatically changed due to the presence of mismatches, we conclude that Watson–Crick base-pairing between crRNA and its target in the Cas10-activating region is sensed by Cas10-Csm as a critical pre-condition for cOA synthesis, albeit in a manner dependent on sequence context.

### Mismatching segments are required in cells for the loss of cOA-mediated interference

**Figure 6.**
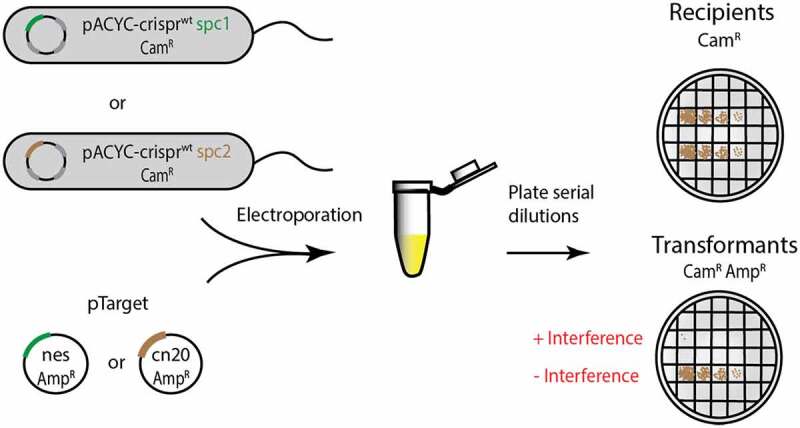
Schematic of the cOA-mediated interference assay. *E. coli* cells harbouring pACYC-*crispr spc1* or pACYC-*crispr spc2* are electroporated with the corresponding pTarget and transformation efficiency (colony-forming units per mL) is scored as a measure of interference. Cells transformed with pTarget have a chloramphenicol-resistant (Cam^R^) and ampicillin-resistant (Amp^R^) phenotype. The pACYC-*crispr* plasmids are labelled as wt because each encodes a catalytically active Csm3 rather than the D32A target RNase-dead variant used for in vitro assays.

In vivo assays of the effect of crRNA–target mismatches on interference are critical towards understanding how Cas10-Csm protects prokaryotes from bacteriophage and mobile genetic elements and also indicating the conditions under which interference can be bypassed, for example by a phage that is genetically divergent from an ancestor targeted by CRISPR. However, interpretation of in vivo interference assays can be challenging because context dictates how much each of Cas10-Csm’s three interference activities contributes to the outcome of the experiment [10–12]. To reduce the number of variables contributing to interference, we re-purposed an *E. coli-*based transformation efficiency assay developed by Terns and co-workers [29,30]. In this assay, *E. coli*-bearing *S. epidermidis* CRISPR-Cas10 on a plasmid with a chloramphenicol marker is challenged by transformation with a plasmid encoding a target of crRNA on a plasmid with an ampicillin marker. In the assay, quantitation of colony-forming units (CFUs) on a Cam plate indicates the number of recipient cells and CFUs on a Cam/Amp plate indicate the number successfully transformed by the target plasmid. Terns and co-workers have shown interference in the assay is only dependent on the cOA-mediated RNase activity of Csm6 which is known to broadly target most RNAs of the cell [30]. We modified the published assay by introducing mismatches into the *nes* target gene and we constructed a pair of new reagents to probe interference in an alternate sequence context: a new pACYC-*crispr* containing strain harbouring the *spc2* gene and a pTarget containing the *cn20* gene (Fig. 6).

**Figure 7.**
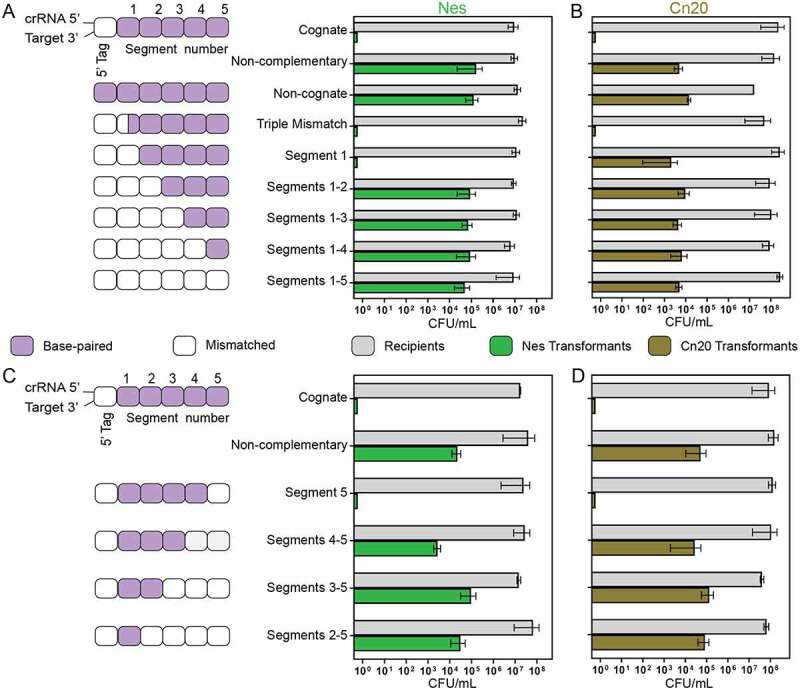
The effect of crRNA–target mismatches on cOA-mediated interference in cells. (A) A schematic of the base-pairing between crRNA and target RNAs encoded in the assay. Mismatching segments were successively introduced into the target gene beginning with segment 1. The effect of mismatched segments on interference was measured by the transformation efficiency of mismatched versions of pTarget harbouring a fragment of the *nes* gene. Recipient colony-forming units (CFUs) indicate the number of cells available for transformation and the transformants CFUs indicate pTarget uptake. (B) The same assay was performed but now pACYC-*crispr^wt^* carried the *spc2* gene which encodes a crRNA targeting the transcript of the *cn20* gene, a fragment of which is encoded on pTarget *cn20*. (C) A schematic of pTarget constructs used in a second interference assay is shown that introduced successive mismatches beginning with segment 5. The assay was performed with pTarget encoding *nes* and (D) pTarget encoding *cn20.*

We wondered to what degree crRNA–target mismatches would affect cOA-mediated interference with the goal of making comparisons between interference in vivo and our in vitro cOA synthesis assays. As before, we were also interested in how sequence context may impact the effect of mismatches on interference. We began by introducing a triple mismatch into the segment 1 coding region of the *nes* gene in pTarget. Whereas, a triple mismatch in vitro reduced cOA synthesis levels so much that they were below the level of accurate quantitation, the triple mismatch at positions +1 to +3 had no effect on interference (Fig. 7A). We then introduced successively mismatching segments into the *nes* gene of pTarget. A fully mismatched *nes* segment 1 also had no effect on interference but if both segments 1 and 2 were mismatched interference receded to background levels. The introduction of mismatches simultaneously into segments 1 to 3, 1 to 4 and 1 to 5 also resulted in no detectable interference (Fig. 7A). We performed the same experiment with pTarget now encoding a fragment of the *cn20* gene and cells carrying *S. epidermidis* CRISPR-Cas10 engineered with a spacer targeting this bacteriophage gene. As observed before, a triple mismatch at positions +1 to +3 in segment 1 had no effect on interference (Fig. 7B). However, if the *cn20* gene was altered so that the entirety of segment 1 was mismatched, interference was dramatically reduced (Fig. 7B). The successive introduction of mismatches in segments 1 to 2, 1 to 3, 1 to 4 and 1 to 5 caused a loss of interference in all cases. The experiments with the *cn20* target, however, revealed a critical distinction between the *nes* and *cn20* sequence contexts with segment 1 being critical to interference in the *cn20* but not *nes* sequence context.

We next performed an experiment designed to test whether, in the cOA-mediated interference assay, Cas10-proximal segments 1 and 2 played a more critical role in interference than segments 3 to 5. That is, interference was lost when segments 1 and 2 were mismatched in *nes* or only segment 1 in *cn20* and we wondered how many segments must be mismatched to inhibit interference if we began successively mismatching segments starting with segment 5 (Fig. 7C). The loss of base-pairing in segment 5 had no effect on interference in either the *nes* or *cn20* sequence contexts; however, the loss of base-pairing in additional segments reduced interference to background levels (Fig. 7C, 7D). Taken together, the two experiments utilizing a *nes* target indicate that the loss of two base-paired segments causes a loss of interference and it does not matter whether the target is mismatched at segments 1 and 2 or 4 and 5. The two experiments in the *cn20* sequence context produced a slightly different result: these experiments indicate that base-pairing in the Cas10-proximal region (including segment 1) has a greater effect on interference than base-pairing in the Cas10-distal region (including segment 5). Loss of base-pairing in segment 1 alone caused a loss of interference in the *cn20* target but when the segments were successively removed from the Cas10-distal region, a loss of base-pairing in segments 4 and 5 were required to abrogate interference.

## Discussion

The effect of mismatches between crRNA and its target on interference by type III systems has been previously investigated but drawing wide-ranging conclusions from these experiments was not possible due to various limitations such as only one target (sequence context) was investigated, mismatches were only examined over a portion of the crRNA–target duplex, or mismatching was investigated in vivo but not with a complementary in vitro assay. By assaying the effects of mismatches on a specific interference activity, cOA-synthesis, across the length of the crRNA–target duplex, in two sequence contexts by complementary approaches, we believe we have filled an important gap in the field.

### Evidence for a Cas10-activating region in positions +1 to +7 of crRNA

**Figure 8.**
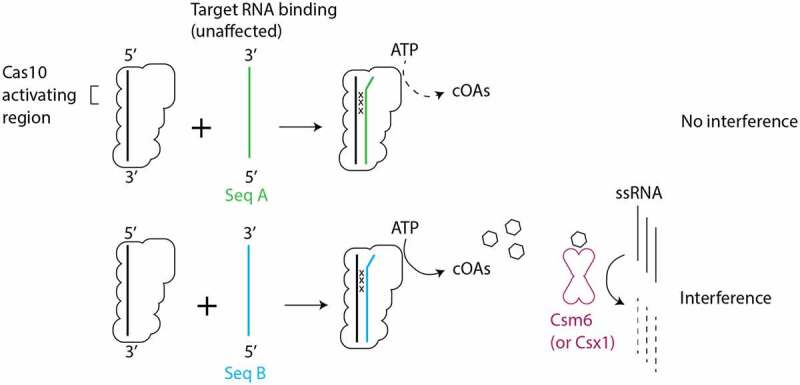
Intrinsic features of the sequence of target RNAs determine the effect of mismatches on cOA-mediated interference. Mismatches between crRNA and target RNA in the Cas10-activating region did not lead to substantial defects in target binding. However, cOA synthesis is sensitive to crRNA–target mismatches in the Cas10-activating region in a manner that depends on intrinsic features of the target sequence. Seq A and Seq B indicate two hypothetical target RNA sequences which differ in their ability to stimulate cOA synthesis. The ‘x’ symbol indicates a crRNA–target RNA mismatch.

It has recently been argued that type III CRISPR systems contain two functionally important regions of crRNA, a Cas10-activating region in the 5’ region of crRNA and a seed sequence in the 3’ region. The Cas10-activating region is located at positions +1 to +7, wherein activation of cOA synthesis is highly sensitive to mismatches [7,25,27]. The in vitro cOA synthesis assays we performed revealed that single mismatches between crRNA and target capable of substantially reducing cOA synthesis were located in +1 to +7 strongly supporting the existence of a Cas10-activating region. Additionally, our affinity binding assays provide a mechanistic clue as to how mismatches in the Cas10-activating region regulate cOA synthesis. The fact that we do not see substantial loss of binding affinity between targets mismatched in segment 1 and crRNA, suggests that correct base-pairing in segment 1 drives a conformational change in Cas10 required for cOA synthesis rather than being critical for tight binding of the target (Fig. 8). Our cell-based interference assays also provide support for the Cas10-activating region’s critical role in interference, albeit modest support. When *nes* was used as the target, similar sensitivity was seen in both the 5’ and 3’ end of the crRNA; mismatches in two contiguous segments on the 5’ end of crRNA (segments 1 and 2) and on the 3’ end of crRNA (segments 4 and 5) were sufficient to eliminate interference.

However, when *cn20* was used as the target mismatches in segment 1 were sufficient for loss of interference, while on the 3’ end of the crRNA loss of pairing in both segments 4 and 5 were required for loss of interference consistent with a special role for positions +1 to +7 in activating cOA synthesis by Cas10. A caveat must be attached to our in vivo interference results that they are performed with recombinantly expressed CRISPR-Cas10 which may affect the amount of cOA synthesized. For example, it may be the case that overexpression causes an abundance of cOA to be made underestimating the effect mismatches have on interference.

RNA-based regulatory systems typically identify their nucleic acid binding partners, among the complex milieu of the cell, by utilizing seed sequences, short nucleotide segments involved in the initial search for complementary partners [31]. For example, Cas9 binds its crRNA so that nucleotides 11–20, which are adjacent to its PAM (protospacer-adjacent-motif) sequence are pre-organized into an A-form helix and displayed towards solvent to facilitate initial binding to its DNA target [][32]. Evidence has been presented for and against the existence of a seed sequence on the 3’ end of cRNA in type III systems [21,24,25,33]. Our cell-based interference experiments did not produce evidence for a 3’ a seed sequence since we did not see heightened sensitivity to mismatches at the 3’ end of crRNA and, in the case where *cn20* was used as the target, observed less sensitivity to mismatches at the 3’ end of crRNA. Pyenson et al. performed a transformation efficiency assay utilizing *S. epidermidis* CRISPR-Cas10, as we did, however the assay was different in several key aspects [21]. These investigators data agree with ours as they also observed the 5’ end of crRNA was more sensitive to mismatches than the 3’ end. The varied findings on the importance of the 3’ region of cRNA in type III systems can be rationalized several ways. It has been shown that target concentration can affect how sensitive type III systems are to mismatches. In a transformation efficiency assay, You et al. showed that pairing from positions +1 to +23 was required for interference when target levels were high but pairing from +1 to +26 was required when target levels were low indicating a greater sensitivity to mismatches at the 3’ end of crRNA when target levels are low. Therefore, varying levels of target among the assays could explain some divergent results. Additionally, it has been argued that the prominent role of pairing at the 3’ end of crRNA is masked by the existence of multiple oligomeric states of type III complexes, a phenomenon that is hard to control for in vivo [25].

### Sequence context affects the sensitivity to mismatches in type III systems

Our work highlights an underappreciated aspect of type III CRISPR systems: conclusions are often drawn from mechanistic experiments performed with a single target sequence. Our results highlight that in vitro and cell-based assays of interference can return different results depending on the target sequence used. Stated differently: intrinsic aspects of the target sequence can affect its ability to stimulate interference (Compare Seq A to Seq B in Fig. 8). Prior to the discovery that cOAs are a critical driver of type III interference, Maniv et al. presented data from assays in staphylococcal cells which hinted that intrinsic aspects of a sequence could affect its sensitivity to mismatches [20]. The investigators used the *nes* and *cn20* genes as model targets, as we did, and found that mismatches at position +2 to +4 of *nes* caused a loss of interference but mismatches at +2 to +4 of *cn20* did not. Surprisingly, the phenomenon depended on gene orientation, whether the gene was on the leading versus lagging strand, which the investigators believed was connected to the fact that the gene was being replicated by rolling circle replication. We have shown intrinsic sequence effects on interference in two assays that differ markedly from those in Maniv et al.: a plasmid transformation efficiency assay that depends on theta replication and an in vitro cOA synthesis assay. Taken together, these data argue that there are several layers of properties that effect how a target sequence will respond to mismatches. Gene orientation is one, but our in vitro results suggest that some target sequences may be less able to induce the conformational changes in Cas10 required for activation as efficiently as others when mismatches occur.

In conclusion, we present data relevant to the ongoing debate over the location and mechanism of specific regions of crRNA that affect interference in type III systems and our data argue for the existence of a Cas10-activating region at positions +1 to +7. Additionally, we present data which argue that the propensity of mismatches within the Cas10-activating region to block interference is an intrinsic feature of the sequence that may vary widely. Resolving the debate over how specific regions of crRNA affect interference is critical since it impacts our understanding of how natural selection drives both mutations in mobile genetic elements to evade type III CRISPR systems and may affect and reinforce the selection of particular spacers by prokaryotes which provide robustness to immunity. Understanding the significance and impact of mismatches in type III systems is also relevant to new biotechnologies being developed with these CRISPR systems [25,27,34,35].

## Materials and methods

### Purification of Cas10-Csm

Cas10-Csm expression and immobilized metal affinity chromatography (IMAC) were performed as previously described [36]. Briefly, overnight cultures of *S. epidermidis* LM1680, which carry the plasmid p*crispr/csm3^D32A^* but lack a genomic *crispr* locus, were used to inoculate 1 L of Brain Heart Infusion media containing appropriate antibiotics. Growth was continued at 37°C until OD_600_ ~ 2.0 was reached and cells were harvested by centrifugation. Lysis was achieved by sonication and the addition of lysostaphin to 28 μg/mL. Following centrifugation, lysate was subjected to IMAC which used wash buffer containing 100 mM NaH_2_PO_4_ pH 8.0, 600 mM NaCl and 40 mM imidazole and elution buffer with the same components except 250 mM imidazole was present. Cas10-Csm-enriched fractions were pooled and layered onto a 5–20% (w/v) sucrose gradient composed of 50 mM Tris HCl pH 8.0, 150 mM NaCl and 5% (v/v) glycerol. Ultracentrifugation was performed for 41 h at 118,000 x *g* on a Beckman SW-32TI rotor. Fractions containing intact Cas10-Csm complex were identified by A_280_ measurements and appeared near the bottom of the gradient consistent with a molecular weight of ~300 kDa. Sample purity was analysed by SDS-PAGE and purified Cas10-Csm was flash-frozen and stored at −80°C.

### Purification and mass spectrometry analysis of crRNAs

CrRNAs were extracted from purified Cas10-Csm^Csm3D32A^ with 1:1 (v/v) ratio of phenol–chloroform–isoamyl alcohol (25:24:1) twice, followed by one extraction with 1 vol. chloroform. For PAGE analysis, crRNAs were ^32^P-labelled by incubation with γ-^32^P-ATP (3000 Ci/mmol) and T4 Polynucleotide kinase at 37°C for 1 h. Extracted crRNAs were mixed 1:1 with formamide dye (5 mM EDTA pH 8.0, 95% formamide v/v) and heated at 70°C for 2 min before loading to a 12% acrylamide, 8 M urea gel. Invitrogen Decade markers were added to the gel to infer crRNA sizes. Electrophoresis was carried out at 50 W for approximately 90 min. Phosphorimaging was performed with storage phosphor screens and a Typhoon FLA 7000 both from GE Healthcare.

For mass spectrometry analysis, crRNAs were desalted with C18 ziptips according to the manufacturer’s instructions. One microlitre of matrix (9 parts of 50 mg/mL 3-hydroxypicolinic acid in 50% acetonitrile/water and 1 part 50 mg/mL ammonium citrate in water) was spotted on a matrix-assisted laser desorption ionization (MALDI) target and was dried by a stream of nitrogen gas. One microlitre of desalted crRNAs was then layered on the dried matrix and the mixture was subjected to MALDI mass spectrometry with a Bruker RapifleX MALDI TOF/TOF.

### cOA synthesis

All reactions were carried out in 50 mM Tris HCl pH 8.0, 150 mM NH_4_Cl, 5% (v/v) glycerol, 500 μM ATP, 30 nM α-^32^P-ATP 3000 Ci/mmol and 10 mM Mg^2+^ (TNG buffer) at 37°C. All reactions were performed with 100 nM Cas10-Csm^Csm3D32A^ and 400 nM target RNA as indicated in TNG buffer for 1-h incubation. cOA reaction products were separated by thin-layer chromatography using previously described methods [28]. Briefly, a chamber containing TLC running buffer composed of 0.2 M ammonium bicarbonate pH 9.3, 70% ethanol and 30% water was pre-warmed to 35°C to saturate the chamber with buffer vapour. Samples were spotted on a silica gel TLC plate 2 cm from the bottom of the plate and were air-dried. The plate was then placed in the chamber for 2 h until the solvent front was approximately 5 cm from the top. The plate was removed from the chamber, dried with a gentle air stream and cOAs were visualized by phosphorimaging.

### Target RNA mismatch library

The target RNA library containing mismatches that were used in cOA synthesis assays is given in supporting information. RNAs are either derived from the sequence of the nickase (*nes*) gene of the pG0400 conjugative plasmid that can be targeted by *spc1* crRNA of p*crispr* [4,37] or derived from the sequence of the *cn20* gene of the staphylococcal phage CNPH82 that can be targeted by *spc2* crRNA. DNA templates (Table S2) used to synthesize the library by *in vitro* transcription were ordered from Eurofins genomics. *In vitro* transcription was performed using the Promega RiboMax kit. Clean-up of the *in vitro* transcription products was performed by DNase I digestion, phenol–chloroform extraction and ethanol precipitation according to the manufacturer’s instructions except phenol–chloroform–isoamyl alcohol 25:24:1 was used for extractions. Synthetic RNAs used in Fig. 3 and Fig. 5 were made by Horizon Discovery. The quality of the RNAs was checked by a 12% acrylamide urea-PAGE run at 300 V for 30 min, stained with SYBR green II (Lonza) and imaged on a Typhoon FLA 7000 imager.

### Affinity binding assays

Varying concentrations of Cas10-Csm^Csm3 D32A^ (8–1024 nM) were incubated with 4 nM ^32^P-radiolabeled ssRNA at 0°C for 30 min. The binding buffer contained 50 mM Tris HCl, pH 8.0, 10 mM Mg^2+^, 5% (v/v) glycerol and 0.5 mM EDTA. Protein-RNA complexes were separated and quantified by a double-membrane binding assay [38]. A sandwich of Amersham Protran 0.45 μm nitrocellulose and Amersham Hybond N+ membranes were soaked in binding buffer and were assembled in a BioRad BioDot vacuum filtration device. Reactions were applied to the membrane sandwich, filtered and the membranes were then washed with 100 μL cold buffer. The membrane sandwich was disassembled, dried and then subjected to phosphorimaging. The fraction bound in each titration series was calculated after integration of the membrane arrays in GE ImageQuant software by dividing the nitrocellulose counts by the sum of the counts from both membranes.

### Interference assays in *E. coli*

A pACYC vector containing a chloramphenicol resistance marker and encoding *S. epidermidis crispr-cas10* for expression in *E. coli* was the gift of Dr Michael Terns of the University of Georgia. The *crispr-cas10* locus is modified to facilitate expression in *E. coli* including the inclusion of T7 promoters, codon optimization of the open reading frames and the inclusion of only a single spacer gene, *spc1* which targets the Nes transcript [29]. We refer to this plasmid as pACYC-*crispr^wt^ spc1* (Fig. 6). This was modified for experiments targeting the *Cn20* transcript: a GeneArt string (ThermoFisher Scientific) encoding *spc2*, was ligated into the plasmid utilizing the NcoI and PspXI sites to create pACYC*-crispr^wt^ spc2* (Fig. 6). This construct was verified by Sanger sequencing with a set of primers that covered the entire plasmid.

A pTrc vector containing an ampicillin marker and a segment of the *nes* gene which we refer to as pTarget-*nes* was also the gift of Dr Michael Terns of the University of Georgia (Fig. 6) [29]. This plasmid was modified by systematically altering segments of the *nes* gene to introduce mismatches between its transcript and crRNA. Mismatches at positions +1, +2 and +3 and +1 to +6 were introduced by QuikChange. Additional *nes* mismatches, segments 1 and 2 or segments 1 to 3, etc., were introduced by Gibson assembly using PCR-linearized pTrc-*nes* and GeneStrands (Eurofins Genomics) [39]. For experiments in which interference was initiated by the *cn20* gene, the *nes* gene of pTrc-*nes* was swapped for the *cn20* gene by Gibson assembly using PCR-linearized vector and a GeneStrand encoding a fragment of *cn20* (Fig. 6). PTrc-*cn20* variants encoding mismatches between the *Cn20* transcript and *spc2-*derived crRNA were constructed by QuikChange (+1, +2 and +3 mismatch and the +1 to +6 mismatch) or by Gibson assembly (segment 1 and 2 or segments 1 to 3 etc.) with GeneStrands. All constructs were verified by Sanger sequencing.

Interference in *E. coli* cells was scored by a transformation efficiency assay. Electrocompetent BL21(DE3) cells harbouring pACYC-*crispr^wt^* plasmids were transformed by electroporation with 40 ng of pTrc-*nes* or pTrc-*cn20*. The transformed cells were then suspended in 1 mL of SOC medium and incubated at 37°C, shaking at 250 rpm for 1 h. Ten-fold serial dilutions were then performed in LB medium and the resulting suspensions were plated on LB-agar plates containing either chloramphenicol (17 µg/mL) or chloramphenicol (17 µg/mL) and ampicillin (50 µg/mL). The plates were incubated overnight at 37°C then colony-forming units per mL (CFU/mL) were quantitated for three replicate transformations of each target plasmid.

## Supplementary Material

Supplemental Material

## Data Availability

PAGE, TLC and membrane (affinity binding) images are available at: https://doi.org/10.5281/zenodo.7041988.
