# Supplementary material for: The effect of crRNA–target mismatches on cOA-mediated interference by a type III-A CRISPR-Cas system
Source: RNA Biol. 2022 Nov 24;19(1):1293–304. doi: 10.1080/15476286.2022.2150812 (PMC9704408; doi:10.1080/15476286.2022.2150812)
Supplement: Supplemental Material [file KRNB_A_2150812_SM1809.docx]

The effect of crRNA-target mismatches on cOA-mediated interference by a type III-A CRISPR-Cas system

Mohamed Nasef^1^, Sarah A. Khweis^1^ and Jack A. Dunkle^1^*

^1^ Department of Chemistry and Biochemistry, University of Alabama, Tuscaloosa, AL, USA

*Corresponding author: Jack A. Dunkle

E-mail: [jadunkle@ua.edu](mailto:jadunkle@ua.edu)

**Running title**: The effect of mismatches on cOA-mediated interference

Supporting Information

**Table S1. Calculated molecular weights of crRNAs versus observed m/z values.**

| **Length (nts)** | **Spc1** | | **Spc2** | | **Spc3** | |
| --- | --- | --- | --- | --- | --- | --- |
|  | Calculated | Observed | Calculated | Observed | Calculated | Observed |
| **37** | 11878.3 | 11879.0 | 11794.1 | 11795.8 | 11803.1 | Not obs. |
| **43** | 13845.5 | 13848.0 | 13715.3 | 13715.6 | 13677.2 | 13676.7 |

The calculated molecular weights listed are average molecular mass assuming 5'OH and 3' OH groups using the Mongo Oligo Mass Calculator v2.06.

**
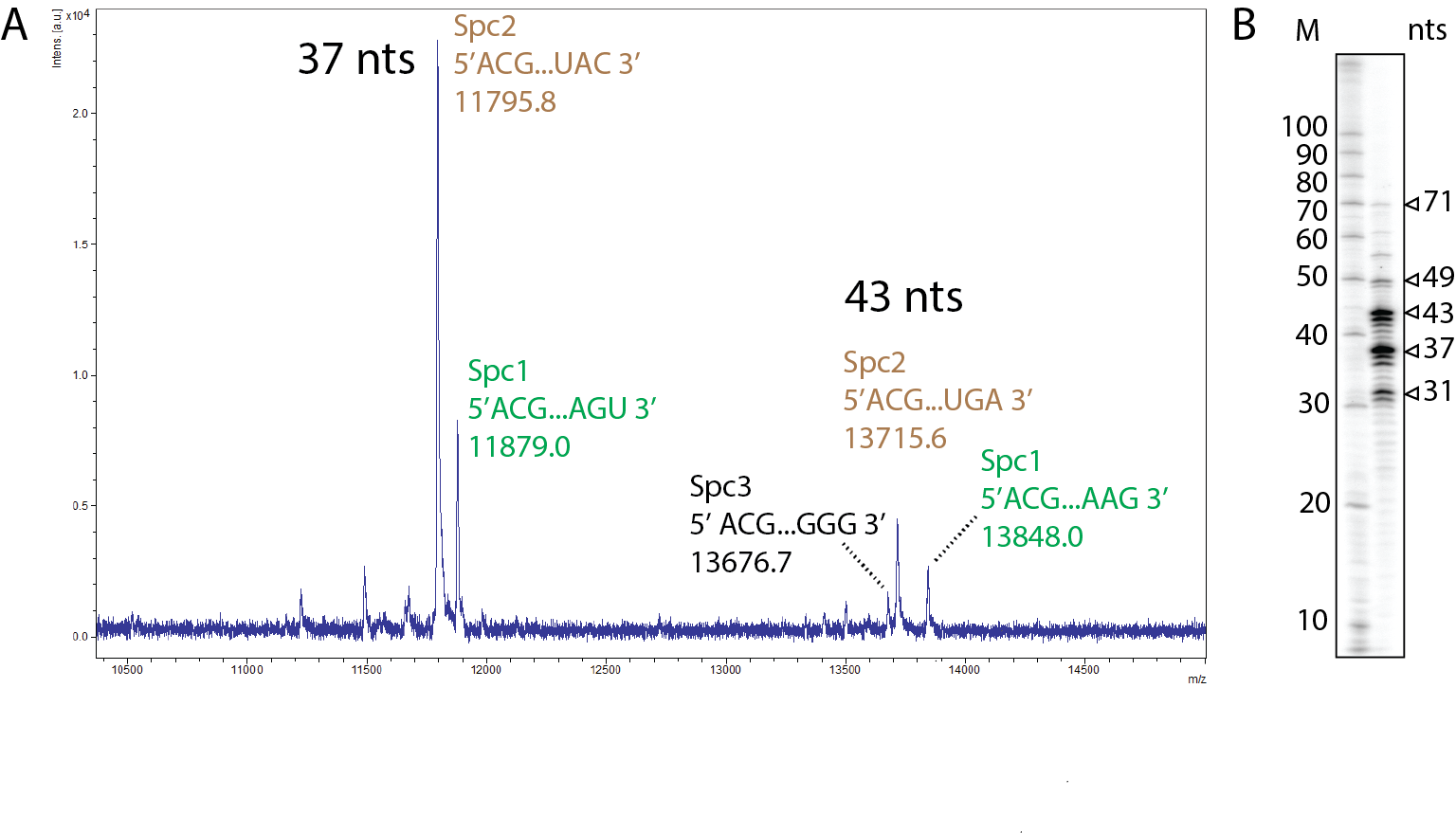
**

**Figure S1. Analyses of crRNA extracted from *S. epidermidis* Cas10-Csm^Csm3 D32A^.** (A) Mass spectrum of 37 and 43 nt crRNAs. (B) A urea-PAGE of ^32^P-labeled crRNAs. M, molecular marker.


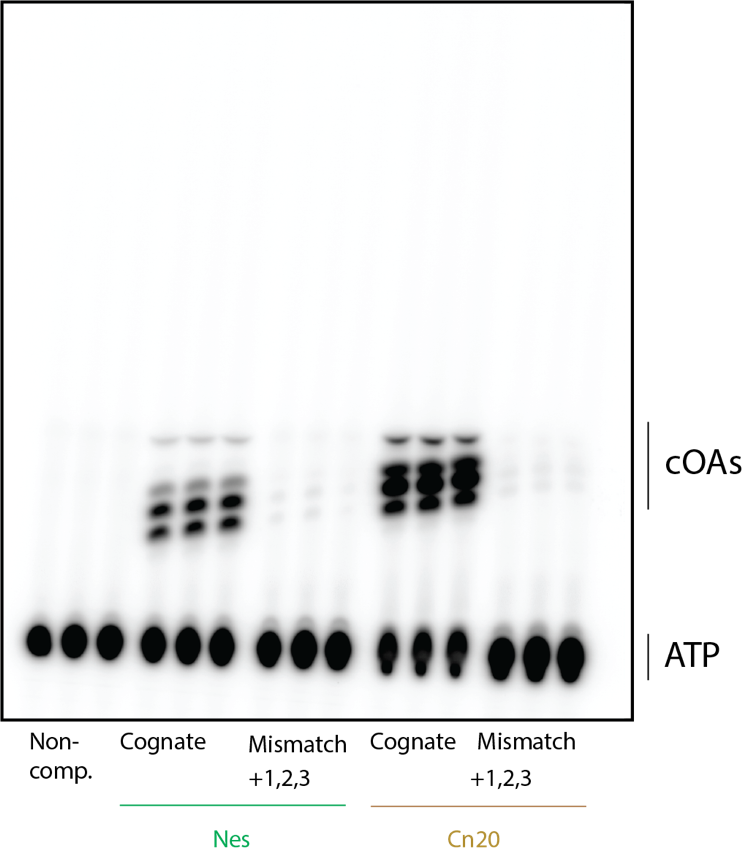


**Figure S2. Uncropped TLC plate displaying the effects of segment 1 triple mismatches on cOA synthesis.**


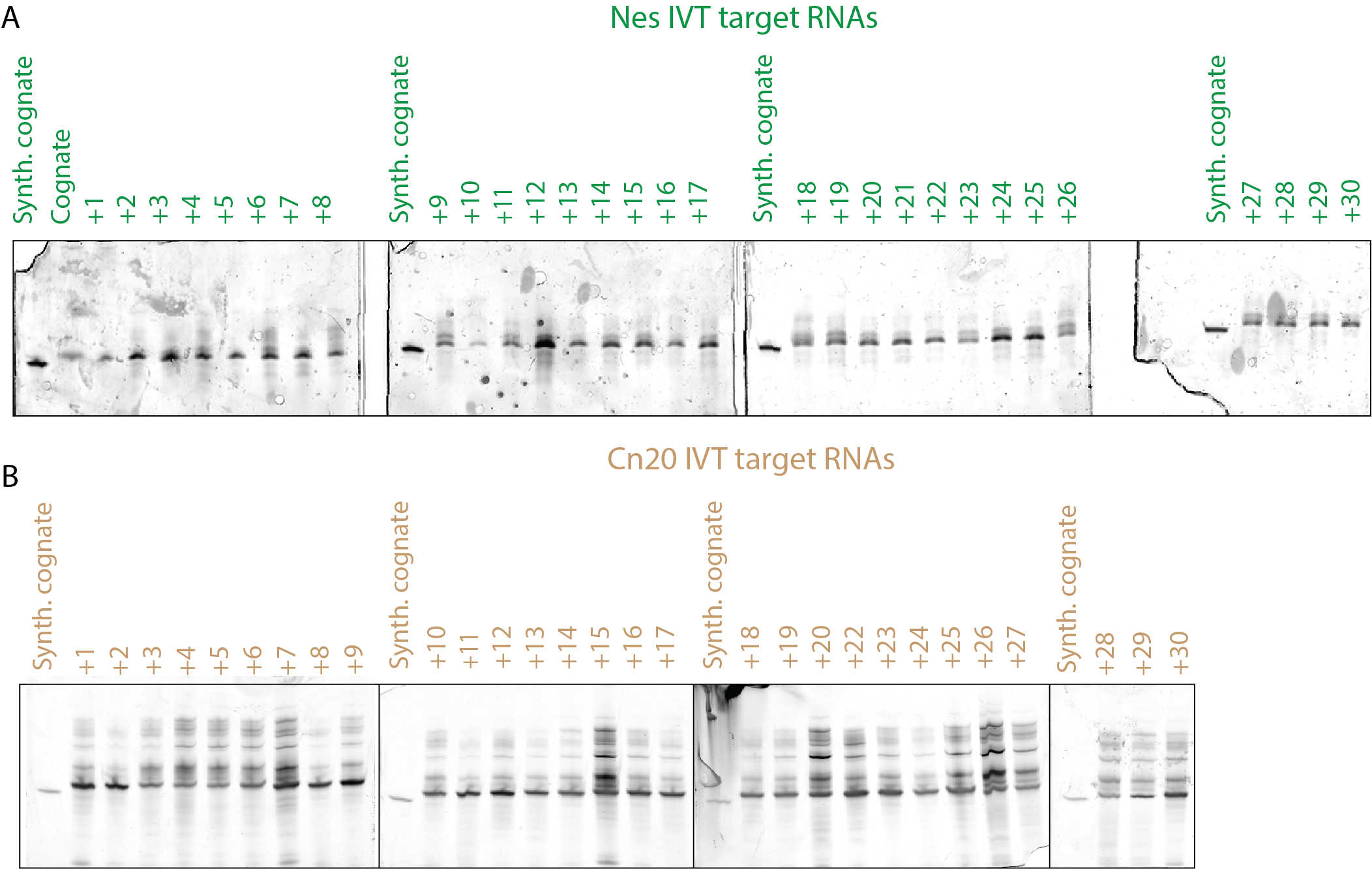


**Figure S3. Urea-PAGE analysis of in vitro transcribed target RNA libraries.**

**
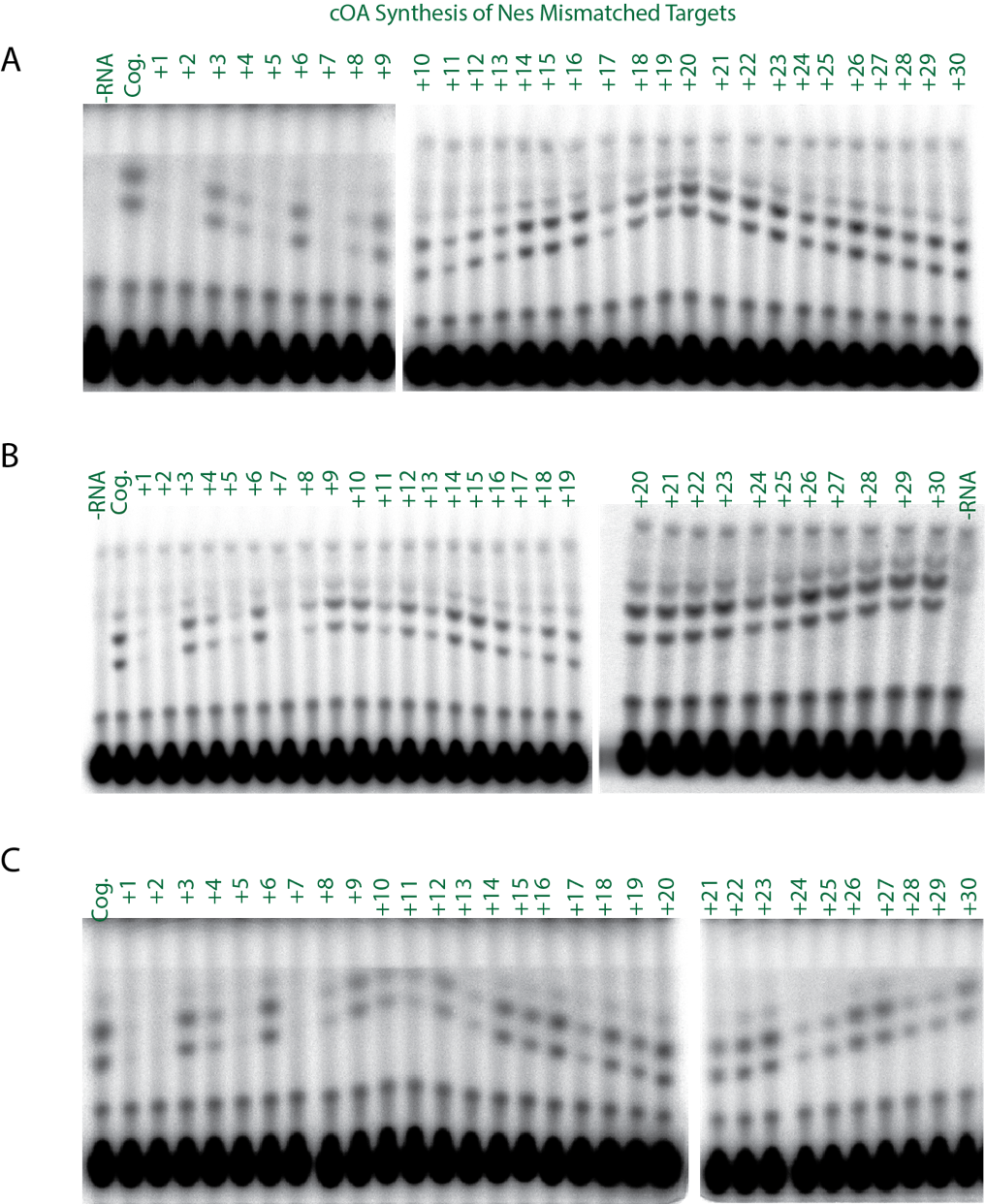
**

**Figure S4. TLC plates for experiments analyzing the effect of single mismatches across positions +1 to +30 of the Nes target RNA on cOA synthesis by Cas10-Csm.**


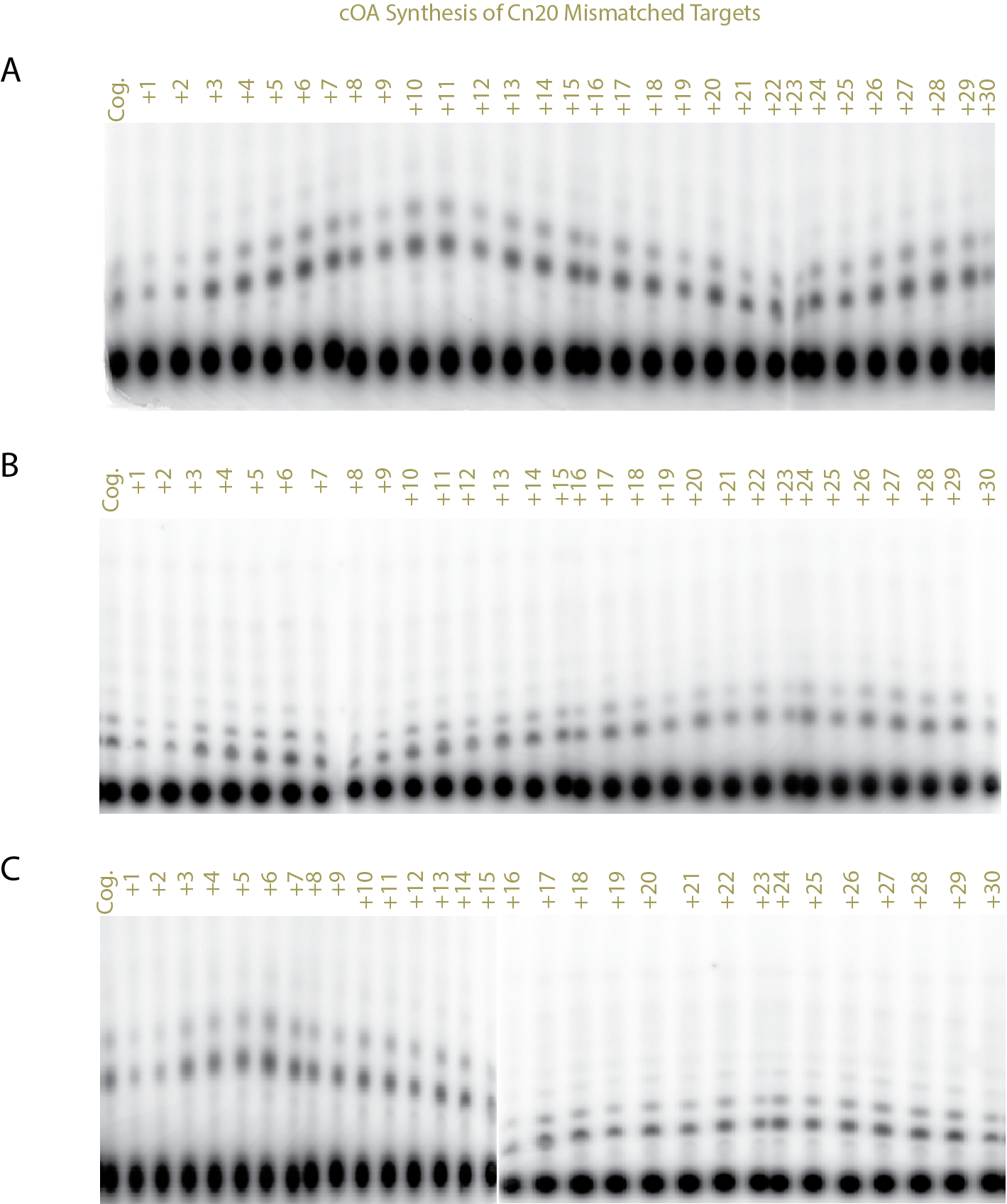


**Figure S5. TLC plates for experiments analyzing the effect of single mismatches across positions +1 to +30 of the Cn20 target RNA on cOA synthesis by Cas10-Csm.**

**
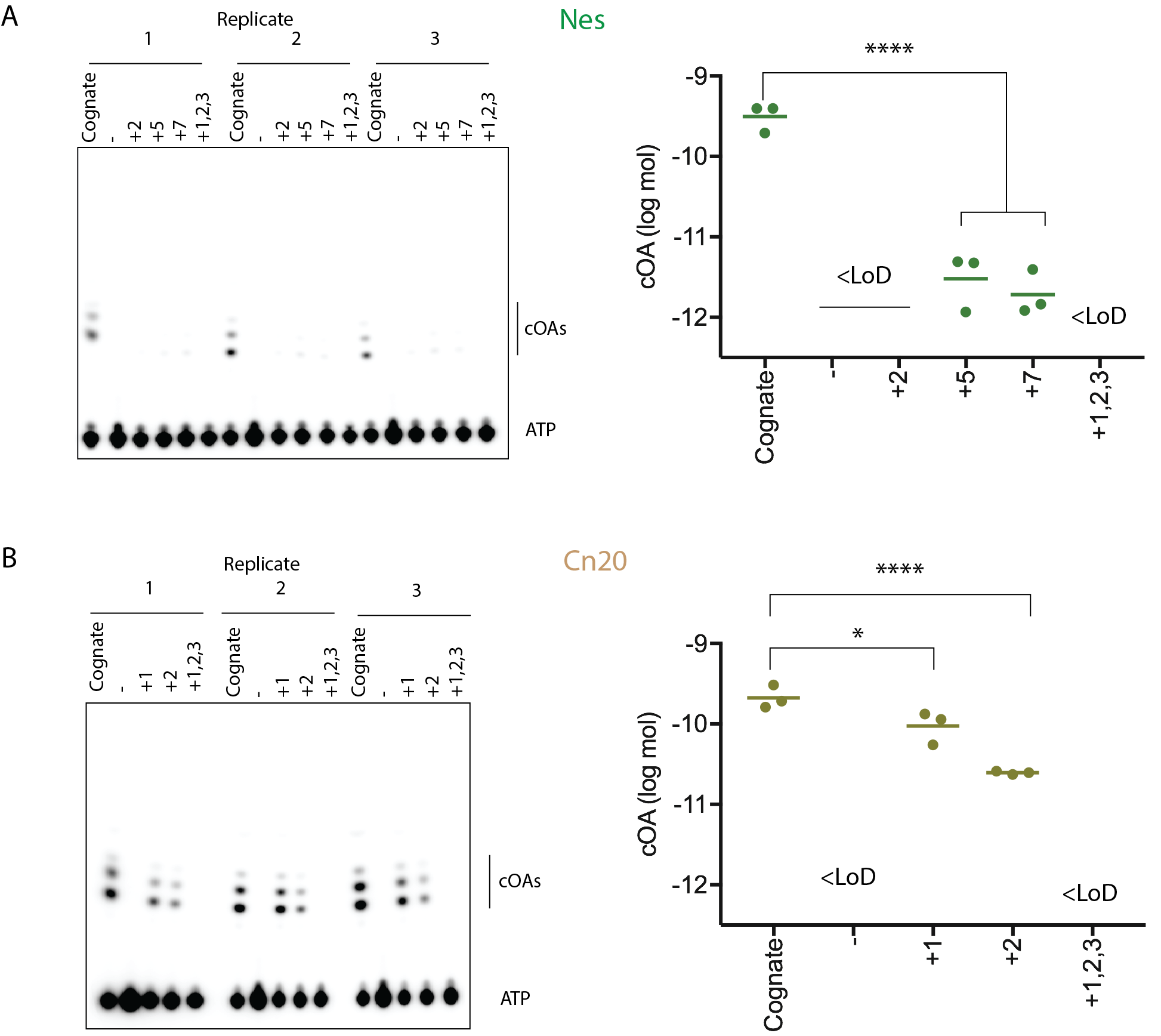
**

**Figure S6. CoA synthesis using chemically synthesized target RNAs.** (A) Synthetic RNAs mimicking the *Nes* target RNA with mismatches as indicated (+1,2,3 indicates a triple mismatch) were used to stimulate cOA synthesis. A reaction (-) without a target RNA was performed as a negative control. The quantitation of the products is shown as a scatter plot. Multiple product amounts were below the limit of detection (<LoD). (B) Synthetic RNAs mimicking the *Cn20* target RNA with mismatches were also used to stimulate cOA synthesis. * P < 0.05, **** P < 0.0001.

**Table S2. Oligos used in the study**

| **Name** | **Sequence** | **Description** |
| --- | --- | --- |
| **Nes_cognate** | CUUUGUACUGAUGAUUUAUAUACUUCGGCAUACGUUCUCUAAA | Nes cognate target RNA |
| **Nes_+1,+2, +3** | CUUUGUACUGAUGAUUUAUAUACUUCGGCAUAGCAUCUCUAAA | Nes target RNA with mismatches at +1, +2, +3 |
| **Nes_+2** | GGCUUUGUACUGAUGAUUUAUAUACUUCGGCAUACCUUCUCUAAA | Nes target RNA with a mismatch at +2 |
| **Nes_+5** | GGCUUUGUACUGAUGAUUUAUAUACUUCGGCAAACGUUCUCUAAA | Nes target RNA with a mismatch at +5 |
| **Nes_+7** | CUUUGUACUGAUGAUUUAUAUACUUCGGGAUACGUUCUCUAAA | Nes target RNA with a mismatch at +7 |
| **Cn20_cognate** | AUCGAUGUAACAUAUGCAAAUGACAAUUAUUACUACGAAGGCG | Cn20 cognate target RNA |
| **Cn20_+1, +2, +3** | AUCGAUGUAACAUAUGCAAAUGACAAUUAUUAGAUCGAAGGCG | Cn20 target RNA with mismatches at +1, +2, +3 |
| **Cn20_+1** | AUCGAUGUAACAUAUGCAAAUGACAAUUAUUACUUCGAAGGCG | Cn20 target RNA with a mismatch at +1 |
| **Cn20_+2** | AUCGAUGUAACAUAUGCAAAUGACAAUUAUUACAACGAAGGCG | Cn20 target RNA with a mismatch at +2 |
| **prMON_900** | GAATTC**TAATACGACTCACTATA**GGCTTTGTACTGATGATTTATATACTTCGGCATACGTTCTCTAAA | Oligo template for IVT producing nes transcript (cognate) |
| **prMON_901** | GAATTC**TAATACGACTCACTATA**GGCTTTGTACTGATGATTTATATACTTCGGCATACGATCTCTAAA | Oligo template for IVT producing a nes transcript with a mismatch at +1 |
| **prMON_902** | GAATTC**TAATACGACTCACTATA**GGCTTTGTACTGATGATTTATATACTTCGGCATACCTTCTCTAAA | Oligo template for IVT producing a nes transcript with a mismatch at +2 |
| **prMON_903** | GAATTC**TAATACGACTCACTATA**GGCTTTGTACTGATGATTTATATACTTCGGCATAGGTTCTCTAAA | Oligo template for IVT producing a nes transcript with a mismatch at +3 |
| **prMON_904** | GAATTC**TAATACGACTCACTATA**GGCTTTGTACTGATGATTTATATACTTCGGCATTCGTTCTCTAAA | Oligo template for IVT producing a nes transcript with a mismatch at +4 |
| **prMON_905** | GAATTC**TAATACGACTCACTATA**GGCTTTGTACTGATGATTTATATACTTCGGCAAACGTTCTCTAAA | Oligo template for IVT producing a nes transcript with a mismatch at +5 |
| **prMON_906** | GAATTC**TAATACGACTCACTATA**GGCTTTGTACTGATGATTTATATACTTCGGCTTACGTTCTCTAAA | Oligo template for IVT producing a nes transcript with a mismatch at +6 |
| **prMON_907** | GAATTC**TAATACGACTCACTATA**GGCTTTGTACTGATGATTTATATACTTCGGGATACGTTCTCTAAA | Oligo template for IVT producing a nes transcript with a mismatch at +7 |
| **prMON_908** | GAATTC**TAATACGACTCACTATA**GGCTTTGTACTGATGATTTATATACTTCGCCATACGTTCTCTAAA | Oligo template for IVT producing a nes transcript with a mismatch at +8 |
| **prMON_909** | GAATTC**TAATACGACTCACTATA**GGCTTTGTACTGATGATTTATATACTTCCGCATACGTTCTCTAAA | Oligo template for IVT producing a nes transcript with a mismatch at +9 |
| **prMON_910** | GAATTC**TAATACGACTCACTATA**GGCTTTGTACTGATGATTTATATACTTGGGCATACGTTCTCTAAA | Oligo template for IVT producing a nes transcript with a mismatch at +10 |
| **prMON_911** | GAATTC**TAATACGACTCACTATA**GGCTTTGTACTGATGATTTATATACTACGGCATACGTTCTCTAAA | Oligo template for IVT producing a nes transcript with a mismatch at +11 |
| **prMON_912** | GAATTC**TAATACGACTCACTATA**GGCTTTGTACTGATGATTTATATACATCGGCATACGTTCTCTAAA | Oligo template for IVT producing a nes transcript with a mismatch at +12 |
| **prMON_913** | GAATTC**TAATACGACTCACTATA**GGCTTTGTACTGATGATTTATATAGTTCGGCATACGTTCTCTAAA | Oligo template for IVT producing a nes transcript with a mismatch at +13 |
| **prMON_914** | GAATTC**TAATACGACTCACTATA**GGCTTTGTACTGATGATTTATATTCTTCGGCATACGTTCTCTAAA | Oligo template for IVT producing a nes transcript with a mismatch at +14 |
| **prMON_915** | GAATTC**TAATACGACTCACTATA**GGCTTTGTACTGATGATTTATAAACTTCGGCATACGTTCTCTAAA | Oligo template for IVT producing a nes transcript with a mismatch at +15 |
| **prMON_916** | GAATTC**TAATACGACTCACTATA**GGCTTTGTACTGATGATTTATTTACTTCGGCATACGTTCTCTAAA | Oligo template for IVT producing a nes transcript with a mismatch at +16 |
| **prMON_917** | GAATTC**TAATACGACTCACTATA**GGCTTTGTACTGATGATTTAAATACTTCGGCATACGTTCTCTAAA | Oligo template for IVT producing a nes transcript with a mismatch at +17 |
| **prMON_918** | GAATTC**TAATACGACTCACTATA**GGCTTTGTACTGATGATTTTTATACTTCGGCATACGTTCTCTAAA | Oligo template for IVT producing a nes transcript with a mismatch at +18 |
| **prMON_919** | GAATTC**TAATACGACTCACTATA**GGCTTTGTACTGATGATTAATATACTTCGGCATACGTTCTCTAAA | Oligo template for IVT producing a nes transcript with a mismatch at +19 |
| **prMON_920** | GAATTC**TAATACGACTCACTATA**GGCTTTGTACTGATGATATATATACTTCGGCATACGTTCTCTAAA | Oligo template for IVT producing a nes transcript with a mismatch at +20 |
| **prMON_921** | GAATTC**TAATACGACTCACTATA**GGCTTTGTACTGATGAATTATATACTTCGGCATACGTTCTCTAAA | Oligo template for IVT producing a nes transcript with a mismatch at +21 |
| **prMON_922** | GAATTC**TAATACGACTCACTATA**GGCTTTGTACTGATGTTTTATATACTTCGGCATACGTTCTCTAAA | Oligo template for IVT producing a nes transcript with a mismatch at +22 |
| **prMON_923** | GAATTC**TAATACGACTCACTATA**GGCTTTGTACTGATCATTTATATACTTCGGCATACGTTCTCTAAA | Oligo template for IVT producing a nes transcript with a mismatch at +23 |
| **prMON_924** | GAATTC**TAATACGACTCACTATA**GGCTTTGTACTGAAGATTTATATACTTCGGCATACGTTCTCTAAA | Oligo template for IVT producing a nes transcript with a mismatch at +24 |
| **prMON_925** | GAATTC**TAATACGACTCACTATA**GGCTTTGTACTGTTGATTTATATACTTCGGCATACGTTCTCTAAA | Oligo template for IVT producing a nes transcript with a mismatch at +25 |
| **prMON_926** | GAATTC**TAATACGACTCACTATA**GGCTTTGTACTCATGATTTATATACTTCGGCATACGTTCTCTAAA | Oligo template for IVT producing a nes transcript with a mismatch at +26 |
| **prMON_927** | GAATTC**TAATACGACTCACTATA**GGCTTTGTACAGATGATTTATATACTTCGGCATACGTTCTCTAAA | Oligo template for IVT producing a nes transcript with a mismatch at +27 |
| **prMON_928** | GAATTC**TAATACGACTCACTATA**GGCTTTGTAGTGATGATTTATATACTTCGGCATACGTTCTCTAAA | Oligo template for IVT producing a nes transcript with a mismatch at +28 |
| **prMON_929** | GAATTC**TAATACGACTCACTATA**GGCTTTGTTCTGATGATTTATATACTTCGGCATACGTTCTCTAAA | Oligo template for IVT producing a nes transcript with a mismatch at +29 |
| **prMON_930** | GAATTC**TAATACGACTCACTATA**GGCTTTGAACTGATGATTTATATACTTCGGCATACGTTCTCTAAA | Oligo template for IVT producing a nes transcript with a mismatch at +30 |
| **prMON_600** | GAATTC**TAATACGACTCACTATA**GGATCGATGTAACATATGCAAATGACAATTATTACTACGAAGGCG | Oligo template for IVT producing a cn20 transcript (cognate) |
| **prMON_601** | GAATTC**TAATACGACTCACTATA**GGATCGATGTAACATATGCAAATGACAATTATTACTTCGAAGGCG | Oligo template for IVT producing a cn20 transcript with a mismatch at +1 |
| **prMON_602** | GAATTC**TAATACGACTCACTATA**GGATCGATGTAACATATGCAAATGACAATTATTACAACGAAGGCG | Oligo template for IVT producing a cn20 transcript with a mismatch at +2 |
| **prMON_603** | GAATTC**TAATACGACTCACTATA**GGATCGATGTAACATATGCAAATGACAATTATTAGTACGAAGGCG | Oligo template for IVT producing a cn20 transcript with a mismatch at +3 |
| **prMON_604** | GAATTC**TAATACGACTCACTATA**GGATCGATGTAACATATGCAAATGACAATTATTTCTACGAAGGCG | Oligo template for IVT producing a cn20 transcript with a mismatch at +4 |
| **prMON_605** | GAATTC**TAATACGACTCACTATA**GGATCGATGTAACATATGCAAATGACAATTATAACTACGAAGGCG | Oligo template for IVT producing a cn20 transcript with a mismatch at +5 |
| **prMON_606** | GAATTC**TAATACGACTCACTATA**GGATCGATGTAACATATGCAAATGACAATTAATACTACGAAGGCG | Oligo template for IVT producing a cn20 transcript with a mismatch at +6 |
| **prMON_607** | GAATTC**TAATACGACTCACTATA**GGATCGATGTAACATATGCAAATGACAATTTTTACTACGAAGGCG | Oligo template for IVT producing a cn20 transcript with a mismatch at +7 |
| **prMON_608** | GAATTC**TAATACGACTCACTATA**GGATCGATGTAACATATGCAAATGACAATAATTACTACGAAGGCG | Oligo template for IVT producing a cn20 transcript with a mismatch at +8 |
| **prMON_609** | GAATTC**TAATACGACTCACTATA**GGATCGATGTAACATATGCAAATGACAAATATTACTACGAAGGCG | Oligo template for IVT producing a cn20 transcript with a mismatch at +9 |
| **prMON_610** | GAATTC**TAATACGACTCACTATA**GGATCGATGTAACATATGCAAATGACATTTATTACTACGAAGGCG | Oligo template for IVT producing a cn20 transcript with a mismatch at +10 |
| **prMON_611** | GAATTC**TAATACGACTCACTATA**GGATCGATGTAACATATGCAAATGACTATTATTACTACGAAGGCG | Oligo template for IVT producing a cn20 transcript with a mismatch at +11 |
| **prMON_612** | GAATTC**TAATACGACTCACTATA**GGATCGATGTAACATATGCAAATGAGAATTATTACTACGAAGGCG | Oligo template for IVT producing a cn20 transcript with a mismatch at +12 |
| **prMON_613** | GAATTC**TAATACGACTCACTATA**GGATCGATGTAACATATGCAAATGTCAATTATTACTACGAAGGCG | Oligo template for IVT producing a cn20 transcript with a mismatch at +13 |
| **prMON_614** | GAATTC**TAATACGACTCACTATA**GGATCGATGTAACATATGCAAATCACAATTATTACTACGAAGGCG | Oligo template for IVT producing a cn20 transcript with a mismatch at +14 |
| **prMON_615** | GAATTC**TAATACGACTCACTATA**GGATCGATGTAACATATGCAAAAGACAATTATTACTACGAAGGCG | Oligo template for IVT producing a cn20 transcript with a mismatch at +15 |
| **prMON_616** | GAATTC**TAATACGACTCACTATA**GGATCGATGTAACATATGCAATTGACAATTATTACTACGAAGGCG | Oligo template for IVT producing a cn20 transcript with a mismatch at +16 |
| **prMON_617** | GAATTC**TAATACGACTCACTATA**GGATCGATGTAACATATGCATATGACAATTATTACTACGAAGGCG | Oligo template for IVT producing a cn20 transcript with a mismatch at +17 |
| **prMON_618** | GAATTC**TAATACGACTCACTATA**GGATCGATGTAACATATGCTAATGACAATTATTACTACGAAGGCG | Oligo template for IVT producing a cn20 transcript with a mismatch at +18 |
| **prMON_619** | GAATTC**TAATACGACTCACTATA**GGATCGATGTAACATATGGAAATGACAATTATTACTACGAAGGCG | Oligo template for IVT producing a cn20 transcript with a mismatch at +19 |
| **prMON_620** | GAATTC**TAATACGACTCACTATA**GGATCGATGTAACATATCCAAATGACAATTATTACTACGAAGGCG | Oligo template for IVT producing a cn20 transcript with a mismatch at +20 |
| **prMON_621** | GAATTC**TAATACGACTCACTATA**GGATCGATGTAACATAAGCAAATGACAATTATTACTACGAAGGCG | Oligo template for IVT producing a cn20 transcript with a mismatch at +21 |
| **prMON_622** | GAATTC**TAATACGACTCACTATA**GGATCGATGTAACATTTGCAAATGACAATTATTACTACGAAGGCG | Oligo template for IVT producing a cn20 transcript with a mismatch at +22 |
| **prMON_623** | GAATTC**TAATACGACTCACTATA**GGATCGATGTAACAAATGCAAATGACAATTATTACTACGAAGGCG | Oligo template for IVT producing a cn20 transcript with a mismatch at +23 |
| **prMON_624** | GAATTC**TAATACGACTCACTATA**GGATCGATGTAACTTATGCAAATGACAATTATTACTACGAAGGCG | Oligo template for IVT producing a cn20 transcript with a mismatch at +24 |
| **prMON_625** | GAATTC**TAATACGACTCACTATA**GGATCGATGTAAGATATGCAAATGACAATTATTACTACGAAGGCG | Oligo template for IVT producing a cn20 transcript with a mismatch at +25 |
| **prMON_626** | GAATTC**TAATACGACTCACTATA**GGATCGATGTATCATATGCAAATGACAATTATTACTACGAAGGCG | Oligo template for IVT producing a cn20 transcript with a mismatch at +26 |
| **prMON_627** | GAATTC**TAATACGACTCACTATA**GGATCGATGTTACATATGCAAATGACAATTATTACTACGAAGGCG | Oligo template for IVT producing a cn20 transcript with a mismatch at +27 |
| **prMON_628** | GAATTC**TAATACGACTCACTATA**GGATCGATGAAACATATGCAAATGACAATTATTACTACGAAGGCG | Oligo template for IVT producing a cn20 transcript with a mismatch at +28 |
| **prMON_629** | GAATTC**TAATACGACTCACTATA**GGATCGATCTAACATATGCAAATGACAATTATTACTACGAAGGCG | Oligo template for IVT producing a cn20 transcript with a mismatch at +29 |
| **prMON_630** | GAATTC**TAATACGACTCACTATA**GGATCGAAGTAACATATGCAAATGACAATTATTACTACGAAGGCG | Oligo template for IVT producing a cn20 transcript with a mismatch at +30 |
| **Cn20 pACYC gene segment** | CACTATAGGGAGACCATGGGATCGATACCCACCCCGAAGAAAAGGGGACGAGAACTAGTAATAATTGTCATTTGCATACGTTACATCGATGATCGATACCCACCCCGAAGAAAAGGGGACGAGAACCTCGAGGCTGTGGTCTAGACATTC | Cn20 ThermoFisher GeneArt |
| **Cn20 pTRC cognate** | GGATCTGTACGACGATGACGATAAGGATCCAACCCTTTTCCAAGCTTATCGATGTAACATATGCAAATGACAATTATTACTACGAAGGCGGCATGCTTCCAAGGCGAATTCGAAGCTTGGCTGTTTTGGCGGATGAGAGAAGATTTTCAGCCTGATACAGATTAAATCAGAAC | Cn20 pTRC cognate |
| **Fully complementary pTRC-Nes** | GGGATCTGTACGACGATGACGATAAGGATCCAACCCTTTTCCAAGCTTCTTTGTACTGATGATTTATATACTTCGGCATACGTGTTCTCGTGCATGCTTCCAAGGCGAATTCGAAGCTTGGCTGTTTTGGCGGATGAGAGAAGATTTTCAGCCTGATACAGATTAAATCA | Complementary 5'-3' tag flank of pTRC- nes |
| **Fully complementary pTRC-Cn20** | GGATCTGTACGACGATGACGATAAGGATCCAACCCTTTTCCAAGCTTATCGATGTAACATATGCAAATGACAATTATTACTAGTTCTCGTGCATGCTTCCAAGGCGAATTCGAAGCTTGGCTGTTTTGGCGGATGAGAGAAGATTTTCAGCCTGATACAGATTAAATCAGAAC | Complementary 5'-3' tag flank of pTRC- cn20 |
| **pr_MON_+1+2+3_F** | CTTGGAAGCATGCTGCTCTTGTGCTATGCCGAAGTATATAAATCATC | Primer for +1, +2, +3 mismatch on pTRC- nes forward |
| **pr_MON_+1+2+3_R** | GATGATTTATATACTTCGGCATAGCACAAGAGCAGCATGCTTCCAAG | Primer for +1, +2, +3 mismatch on pTRC- nes reverse |
| **pr_SAK_001** | TGGAAGCATGCTGCTCTTGTGCATAGCCGAAGTATATAAATCATCAG | Primer for +1 to +6 mismatch on pTRC- nes |
| **pr_SAK_002** | CTGATGATTTATATACTTCGGCTATGCACAAGAGCAGCATGCTTCCA | Primer for +1 to +6 mismatch on pTRC- nes |
| **pr_SAK_020** | GAAGCATGCCGCCTTCGATCTAATAATTGTCATTTGCATATGTTACATCGATA | Primer for +1, +2, +3 mismatch on pTRC- cn20 |
| **pr_SAK_021** | TATCGATGTAACATATGCAAATGACAATTATTAGATCGAAGGCGGCATGCTTC | Primer for +1, +2, +3 mismatch on pTRC- cn20 |
| **Segments 1-2 Nes** | GGGATCTGTACGACGATGACGATAAGGATCCAACCCTTTTCCAAGCTTCTTTGTACTGATGATTTATATACAAGCCGTATGCACAAGAGCAGCATGCTTCCAAGGCGAATTCGAAGCTTGGCTGTTTTGGCGGATGAGAGAAGATTTTCAGCCTGATACAGATTAAATCA | Mismatch of segments 1-2 on pTRC-nes |
| **Segments 1-3 Nes** | GGGATCTGTACGACGATGACGATAAGGATCCAACCCTTTTCCAAGCTTCTTTGTACTGATGATTTTATATGAAGCCGTATGCACAAGAGCAGCATGCTTCCAAGGCGAATTCGAAGCTTGGCTGTTTTGGCGGATGAGAGAAGATTTTCAGCCTGATACAGATTAAATCA | Mismatch of segments 1-3 on pTRC-nes |
| **Segments 1-4 Nes** | GGGATCTGTACGACGATGACGATAAGGATCCAACCCTTTTCCAAGCTTCTTTGTACTGAACTAAATATATGAAGCCGTATGCACAAGAGCAGCATGCTTCCAAGGCGAATTCGAAGCTTGGCTGTTTTGGCGGATGAGAGAAGATTTTCAGCCTGATACAGATTAAATCA | Mismatch of segments 1-4 on pTRC-nes |
| **Segments 1-5 Nes** | GGGATCTGTACGACGATGACGATAAGGATCCAACCCTTTTCCAAGCTTCTTTGATGACTACTAAATATATGAAGCCGTATGCACAAGAGCAGCATGCTTCCAAGGCGAATTCGAAGCTTGGCTGTTTTGGCGGATGAGAGAAGATTTTCAGCCTGATACAGATTAAATCA | Mismatch of segments 1-5 on pTRC-nes |
| **Segment 5 Nes** | GGGATCTGTACGACGATGACGATAAGGATCCAACCCTTTTCCAAGCTTCTTTGATGACTTGATTTATATACTTCGGCATACGTCAAGAGCAGCATGCTTCCAAGGCGAATTCGAAGCTTGGCTGTTTTGGCGGATGAGAGAAGATTTTCAGCCTGATACAGATTAAATCA | Mismatch of segment 5 on pTRC-nes |
| **Segments 4-5 Nes** | GGGATCTGTACGACGATGACGATAAGGATCCAACCCTTTTCCAAGCTTCTTTGATGACTACTAAAATATACTTCGGCATACGTCAAGAGCAGCATGCTTCCAAGGCGAATTCGAAGCTTGGCTGTTTTGGCGGATGAGAGAAGATTTTCAGCCTGATACAGATTAAATCA | Mismatch of segments 4-5 on pTRC-nes |
| **Segments 3-5 Nes** | GGGATCTGTACGACGATGACGATAAGGATCCAACCCTTTTCCAAGCTTCTTTGATGACTACTAAATATATGTTCGGCATACGTCAAGAGCAGCATGCTTCCAAGGCGAATTCGAAGCTTGGCTGTTTTGGCGGATGAGAGAAGATTTTCAGCCTGATACAGATTAAATCA | Mismatch of segments 3-5 on pTRC-nes |
| **Segments 2-5 Nes** | GGGATCTGTACGACGATGACGATAAGGATCCAACCCTTTTCCAAGCTTCTTTGATGACTACTAAATATATGAAGCCGATACGTCAAGAGCAGCATGCTTCCAAGGCGAATTCGAAGCTTGGCTGTTTTGGCGGATGAGAGAAGATTTTCAGCCTGATACAGATTAAATCA | Mismatch of segments 2-5 on pTRC-nes |
| **Segment 1 Cn20** | GGATCTGTACGACGATGACGATAAGGATCCAACCCTTTTCCAAGCTTATCGATGTAACATATGCAAATGACAATTAAATGATCGAAGGCGGCATGCTTCCAAGGCGAATTCGAAGCTTGGCTGTTTTGGCGGATGAGAGAAGATTTTCAGCCTGATACAGATTAAATCAGAAC | Mismatch of segment 1 on pTRC-cn20 |
| **Segments 1-2 Cn20** | GGATCTGTACGACGATGACGATAAGGATCCAACCCTTTTCCAAGCTTATCGATGTAACATATGCAAATGAGTTAATAATGATCGAAGGCGGCATGCTTCCAAGGCGAATTCGAAGCTTGGCTGTTTTGGCGGATGAGAGAAGATTTTCAGCCTGATACAGATTAAATCAGAAC | Mismatch of segments 1-2 on pTRC-cn20 |
| **Segments 1-3 Cn20** | GGATCTGTACGACGATGACGATAAGGATCCAACCCTTTTCCAAGCTTATCGATGTAACATATGCTTTACTGTTAATAATGATCGAAGGCGGCATGCTTCCAAGGCGAATTCGAAGCTTGGCTGTTTTGGCGGATGAGAGAAGATTTTCAGCCTGATACAGATTAAATCAGAAC | Mismatch of segments 1-3 on pTRC-cn20 |
| **Segments 1-4 Cn20** | GGATCTGTACGACGATGACGATAAGGATCCAACCCTTTTCCAAGCTTATCGATGTAACTATACGTTTACTGTTAATAATGATCGAAGGCGGCATGCTTCCAAGGCGAATTCGAAGCTTGGCTGTTTTGGCGGATGAGAGAAGATTTTCAGCCTGATACAGATTAAATCAGAAC | Mismatch of segments 1-4 on pTRC-cn20 |
| **Segment 1-5 Cn20** | GGATCTGTACGACGATGACGATAAGGATCCAACCCTTTTCCAAGCTTATCGAACATTGTATACGTTTACTGTTAATAATGATCGAAGGCGGCATGCTTCCAAGGCGAATTCGAAGCTTGGCTGTTTTGGCGGATGAGAGAAGATTTTCAGCCTGATACAGATTAAATCAGAAC | Mismatch of segments 1-5 on pTRC-cn20 |
| **Segment 5 Cn20** | GGATCTGTACGACGATGACGATAAGGATCCAACCCTTTTCCAAGCTTATCGAACATTGGTATGCAAATGACAATTATTACTACGAAGGCGGCATGCTTCCAAGGCGAATTCGAAGCTTGGCTGTTTTGGCGGATGAGAGAAGATTTTCAGCCTGATACAGATTAAATCAGAAC | Mismatch of segment 5 on pTRC-cn20 |
| **Segments 4-5 Cn20** | GGATCTGTACGACGATGACGATAAGGATCCAACCCTTTTCCAAGCTTATCGAACATTGCATACGAAATGACAATTATTACTACGAAGGCGGCATGCTTCCAAGGCGAATTCGAAGCTTGGCTGTTTTGGCGGATGAGAGAAGATTTTCAGCCTGATACAGATTAAATCAGAAC | Mismatch of segments 4-5 on pTRC-cn20 |
| **Segments 3-5 Cn20** | GGATCTGTACGACGATGACGATAAGGATCCAACCCTTTTCCAAGCTTATCGAACATTGCATACGTTTACTCAATTATTACTACGAAGGCGGCATGCTTCCAAGGCGAATTCGAAGCTTGGCTGTTTTGGCGGATGAGAGAAGATTTTCAGCCTGATACAGATTAAATCAGAAC | Mismatch of segments 3-5 on pTRC-cn20 |
| **Segments 2-5 Cn20** | GGATCTGTACGACGATGACGATAAGGATCCAACCCTTTTCCAAGCTTATCGAACATTGCATACGTTTACTGTTAATTTACTACGAAGGCGGCATGCTTCCAAGGCGAATTCGAAGCTTGGCTGTTTTGGCGGATGAGAGAAGATTTTCAGCCTGATACAGATTAAATCAGAAC | Mismatch of segments 2-5 on pTRC-cn20 |

Bold type indicates a T7 promoter region. In vitro transcription was performed using the DNA oligo templates given above with each annealed to an oligo with the reverse complement sequence (not shown).
